# Intentions poorly explain how and why people engage in offensive and defensive forms of violence

**DOI:** 10.1073/pnas.2611331123

**Published:** 2026-05-22

**Authors:** Angelo Romano, Carsten K. W. De Dreu

**Affiliations:** ^a^ Social, Economic, and Organisational Psychology Department, Leiden University, Leiden 2313 BP, the Netherlands; ^b^ Faculty of Economics and Business, Groningen University, Groningen 9747 AE, the Netherlands; ^c^ Faculty of Behavioral and Social Sciences, University of Groningen, Groningen 9712 TS, the Netherlands; ^d^ Behavioral Ecology and Sociobiology Unit, German Primate Center; Leibniz Institute for Primate Research, Göttingen 37077, Germany

Kunst et al. distinguish between offensive and defensive forms of violent extremism (1), arguing that these represent psychologically separable motivational systems with distinct individual and societal correlates. Their contribution adds to evolutionary anthropology (2) and behavioral game-theory (3) yet leaves open whether and how self-reported intentions translate into behavioral strategies for offensive and defensive conflict. In fact, earlier work using intergroup contest games (3–5) suggest that some of the conclusions in Kunst et al. may need revision or further testing.

A first concern pertains to the psychological origins of intentions to engage in defensive and offensive violence. Kunst et al. attribute the higher prevalence of defensive intentions primarily to their greater moral legitimacy. However, a similar asymmetry emerges in incentivized behavioral settings in which moral considerations are minimized, and payoffs are explicitly defined (6, 7). In such settings, individuals invest more in defense than in offensive attack because defensive investments reduce potential losses while offensive investments entail riskier attempts to appropriate resources. From this perspective, both intentions and behavior emerge from the underlying payoff structure of conflict—the observed predominance of defensive intentions in Kunst et al. may reflect, at least in part, strategic considerations rather than considerations of moral legitimacy (5).

Second, whether and how intentions translate into behavior depends on expectations individuals have about the behavioral intentions of those within their own groups and societies (8), and those held by out-groups and hostile enemies (9). The problem is that expectations about offensive and defensive violence are often misconstrued across groups (10). For example, participants across 51 countries consistently failed to accurately anticipate how competitive foreigners were, leading to widespread over- and underinvestment in defensive actions. Strikingly, individuals tended to invest more in defense against groups that were objectively less competitive and less against those that were more competitive. Without accounting for expectations, it is difficult to infer whether (self-reported) intentions to act violently are driven by dominance-seeking, threat-avoidance, strategic miscalibration, or some combination thereof.

**Fig. 1.**
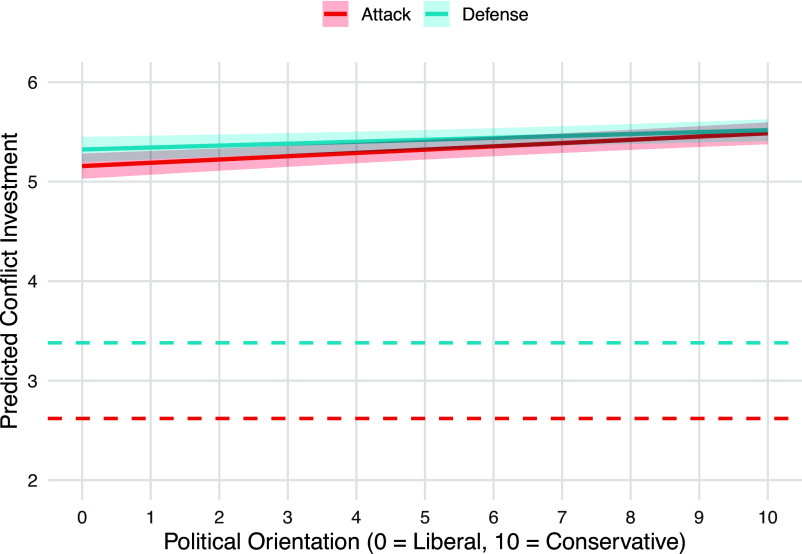
Political orientation and investing in attack and defense against foreigners across 51 societies. Dashed lines indicate investment levels when played in equilibrium (for detail see refs. 6 and 10).

Third and finally, Kunst et al. report that liberal political identification is associated with higher (lower) intentions for offensive (defensive) violence, a pattern they interpret in terms of system-challenging vs. system-preserving motivations. New analyses of ref. 10 suggest a different pattern when it comes to behavior: individuals with more conservative orientations invest more in offensive attack (b = 0.03, *P* < 0.001) and in defense (b = 0.02, *P* = 0.005) (Fig. 1). This divergence between self-reported intentions in ref. 1 and strategic decisions in ref. 10 resonates with the possibility that ideology shapes how individuals interpret and justify their actions, rather than driving behavioral decision-making in conflict.

While the distinction between offensive and defensive motivations is appreciated (2, 3), the exclusive reliance on self-reports and concurrent measurement in Kunst et al. prohibits causal inferences about its origins, dynamics, and consequences. Incentivized experiments of intergroup conflict suggest, indeed, that self-interest and beliefs about enemy hostility may be foundational to offensive and defensive violence, and that moral considerations follow rather than precede individual propensity for violence.
